# Potential impact of climate change on the geographical distribution of two wild vectors of Chagas disease in Chile: *Mepraia spinolai* and *Mepraia gajardoi*

**DOI:** 10.1186/s13071-019-3744-9

**Published:** 2019-10-14

**Authors:** Rubén Garrido, Antonella Bacigalupo, Francisco Peña-Gómez, Ramiro O. Bustamante, Pedro E. Cattan, David E. Gorla, Carezza Botto-Mahan

**Affiliations:** 1 Departamento de Ciencias Ecológicas, Facultad de Ciencias, Universidad de Chile, Casilla 653, Santiago, Chile; 2 Departamento de Biología y Química, Facultad de Ciencias Básicas, Universidad Católica del Maule, Talca, Chile; 3 Departamento de Ciencias Biológicas Animales, Facultad de Ciencias Veterinarias y Pecuarias, Universidad de Chile, Casilla 2, Correo 15, Santiago, Chile; 4 Instituto de Diversidad y Ecología Animal, CONICET-Universidad Nacional de Córdoba, Córdoba, Argentina

**Keywords:** Triatominae, Wild vectors, *Trypanosoma cruzi*, Climate change, Maxent, Species distribution models, Climate suitability, Future scenarios, Kissing bugs

## Abstract

**Background:**

*Mepraia gajardoi* and *Mepraia spinolai* are endemic triatomine vector species of *Trypanosoma cruzi*, a parasite that causes Chagas disease. These vectors inhabit arid, semiarid and Mediterranean areas of Chile. *Mepraia gajardoi* occurs from 18° to 25°S, and *M. spinolai* from 26° to 34°S. Even though both species are involved in *T. cruzi* transmission in the Pacific side of the Southern Cone of South America, no study has modelled their distributions at a regional scale. Therefore, the aim of this study is to estimate the potential geographical distribution of *M. spinolai* and *M. gajardoi* under current and future climate scenarios.

**Methods:**

We used the Maxent algorithm to model the ecological niche of *M. spinolai* and *M. gajardoi*, estimating their potential distributions from current climate information and projecting their distributions to future climatic conditions under representative concentration pathways (RCP) 2.6, 4.5, 6.0 and 8.5 scenarios. Future predictions of suitability were constructed considering both higher and lower public health risk situations.

**Results:**

The current potential distributions of both species were broader than their known ranges. For both species, climate change projections for 2070 in RCP 2.6, 4.5, 6.0 and 8.5 scenarios showed different results depending on the methodology used. The higher risk situation showed new suitable areas, but the lower risk situation modelled a net reduction in the future potential distribution areas of *M. spinolai* and *M. gajardoi*.

**Conclusions:**

The suitable areas for both species may be greater than currently known, generating new challenges in terms of vector control and prevention. Under future climate conditions, these species could modify their potential geographical range. Preventive measures to avoid accidental human vectorial transmission by wild vectors of *T. cruzi* become critical considering the uncertainty of future suitable areas projected in this study.

## Background

Chagas disease, or American trypanosomiasis, is an endemic vector-borne disease affecting between six and eight million people, with an attributed number of deaths of approximately 12,000 per year worldwide [1]. Its causative agent is the protozoan *Trypanosoma cruzi*, transmitted by hematophagous insects of the subfamily Triatominae to mammals [2]. In Chile, the current human prevalence of Chagas disease is 0.7%, with 0.6% and 1.5% in urban and rural zones, respectively [3]. The vector species present in Chile are *Triatoma infestans*, *Mepraia gajardoi*, *M. parapatrica* and *M. spinolai* [4]. These triatomines occur in rural and suburban zones from 18°30′S to 34°36′S [5].

*Mepraia gajardoi* is currently detected on the northern coastal zones where the arid climate is common, whilst *M. spinolai* can be found in valleys in the Mediterranean-semiarid climate zones [6]. The domestic vectorial transmission by the domestic vector *T. infestans* was declared interrupted in 1999; however, wild vectors are still a problem in rural areas of Chile [3]. The sylvatic *M. spinolai* is very abundant in stone quarries of periurban zones [7] where it feeds on wild rodents, goats, dogs, cats, rabbits and humans [8, 9], and several home invasion complaints are notified to the authorities every year (data requested from http://www.portaltransparencia.cl). *Mepraia gajardoi* is abundant near seaweed collector settlements, where it preferably feeds on sea birds, marine mammals, lizards, dogs, cats and humans [10, 11]. These situations are epidemiologically relevant, especially considering that the prevalence of *T. cruzi* in *M. spinolai* populations can reach up to 76.1% [12] and 27.0% for *M. gajardoi* [13]. Under this scenario, it is necessary to explore potential areas where these species can be detected, increasing information on habitat preferences, niche requirements and geographical distributions.

In general, a disease transmission system is composed of a set of species that interact in hosting and vectoring a pathogen in space [14], and all the component species have a unique biogeography related to its ecological niche (i.e. those conditions appropriate for its survival and reproduction), interactions with other species and accessible areas over time [14, 15]. Therefore, a large part of disease transmission risk corresponds to the intersection of the distributional areas of the species involved [14].

Species distribution models (SDMs) are proper tools to examine the potential geographical distribution of species [16]. These models correlate the georeferenced occurrences of a species with environmental information (for instance, climatic information), identifying suitable areas for the survival of its populations given its niche requirements [17]. Climate is key to understanding the geographical distribution of species at a large spatial scale [18–20]. Niche conservation, i.e. the tendency of species to maintain their niche requirements over space and time [21, 22], is frequently assumed in SDMs. If the niche is conserved, it is possible to project the niche requirements to other geographical regions or to other times, past or future [21, 22]. Under this assumption, SDMs have been extended to the study of infectious diseases and host, parasite, reservoir and/or vector modelling [20, 23]. For example, the geographical distributions of West Nile virus vectors have been modelled [24], the spatial dynamics of dengue vectors and human dengue cases [25], among many others. Chagas disease risk has also been modelled [26, 27], as well as the reservoirs of *T. cruzi* [28] and some of its vectors [29–36]. SDMs are also useful to project the potential distribution under climate change conditions, which could help to identify spatial changes of infectious diseases [37, 38]. Modelling under future climate conditions can be important for making decisions about control and disease surveillance, anticipating appropriate measures [39]. The aim of this study is to estimate the potential geographical distribution of two wild vectors of Chagas disease in Chile, *M. spinolai* and *M. gajardoi*, under current and future climate scenarios.

## Methods

### Study areas

This study was carried out considering the current distribution of both *Mepraia* species that includes desert, matorral and steppe ecoregions in the Pacific side of the Southern Cone of South America [40] (Additional file 1: Figure S1, modified from [40]). We chose this criterion because the wild vectors of Chagas disease included in this study are considered endemic for those ecoregions [4].

### Triatomine species and occurrence data

We only used georeferenced occurrences data to allow their association with environmental data (Additional file 2: Datasets S1 and S2). Our sources were: field databases collected by different research groups between 2008–2016 (unpublished data); 19 published scientific articles with georeferenced locations (Additional file 3: Table S1); collections of the Museo Nacional de Historia Natural (Santiago, Chile) and the Museo Entomológico de la Universidad Metropolitana de Ciencias de la Educación (Santiago, Chile); and reported sightings of triatomines corresponding to notifications of house or peridomicile intrusion informed by rural communities to public health centres, which included the insect specimen, obtained through the governmental website (http://www.portaltransparencia.cl). All specimens were identified to the species level by researchers with entomological training using taxonomical keys [4, 6]. The compiled database included 790 occurrences for *M. spinolai* and 19 for *M. gajardoi.* Geographical duplicated occurrences, considering 1 km^2^ for each occurrence point, were removed from the database using NicheToolBox (http://shiny.conabio.gob.mx:3838/nichetoolb2/), so the available database for modelling included 151 occurrences for *M. spinolai* and 13 for *M. gajardoi* (Additional file 2: Datasets S1 and S2).

### Environmental data

We used climate data from WorldClim v.1.4 because it includes both current and future climate conditions (http://worldclim.org/version1) [41]. We selected a subset of five of the 19 available climate variables within the WorldClim dataset, based on expert knowledge of the biology of these vectors [27, 42–44]. In the models, we included annual trends (Bio12: annual precipitation), seasonality (Bio7: temperature annual range) and limiting or extreme environmental factors (Bio2: mean temperature diurnal range; Bio10: mean temperature of warmest quarter; Bio11: mean temperature of coldest quarter) [27]. We used the data at 30 arc-second spatial resolution (approximately 1 km^2^ at the equator).

**Fig. 1:**
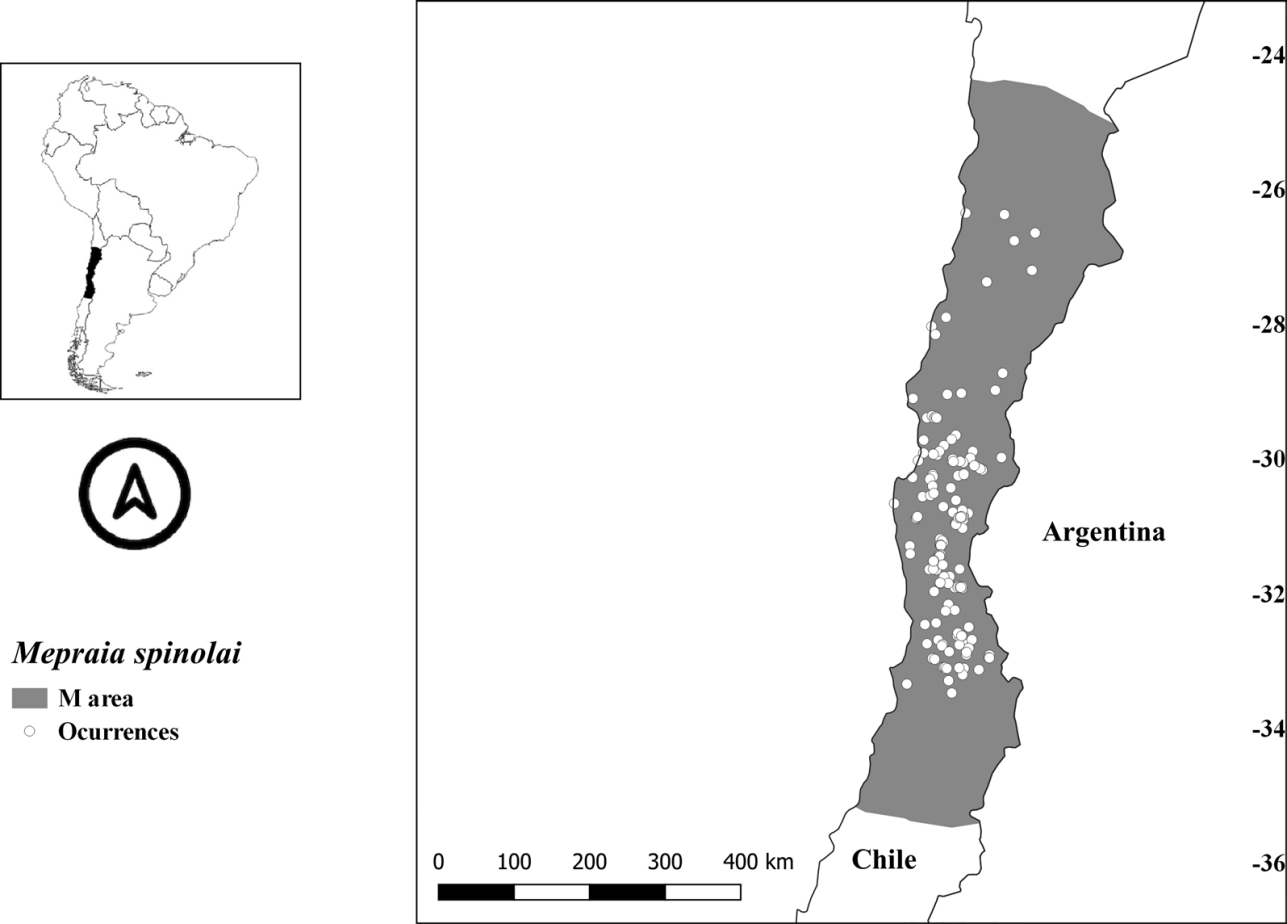
Accessible areas (M) and occurrences (open circles) used to model *Mepraia spinolai*

**Fig. 2:**
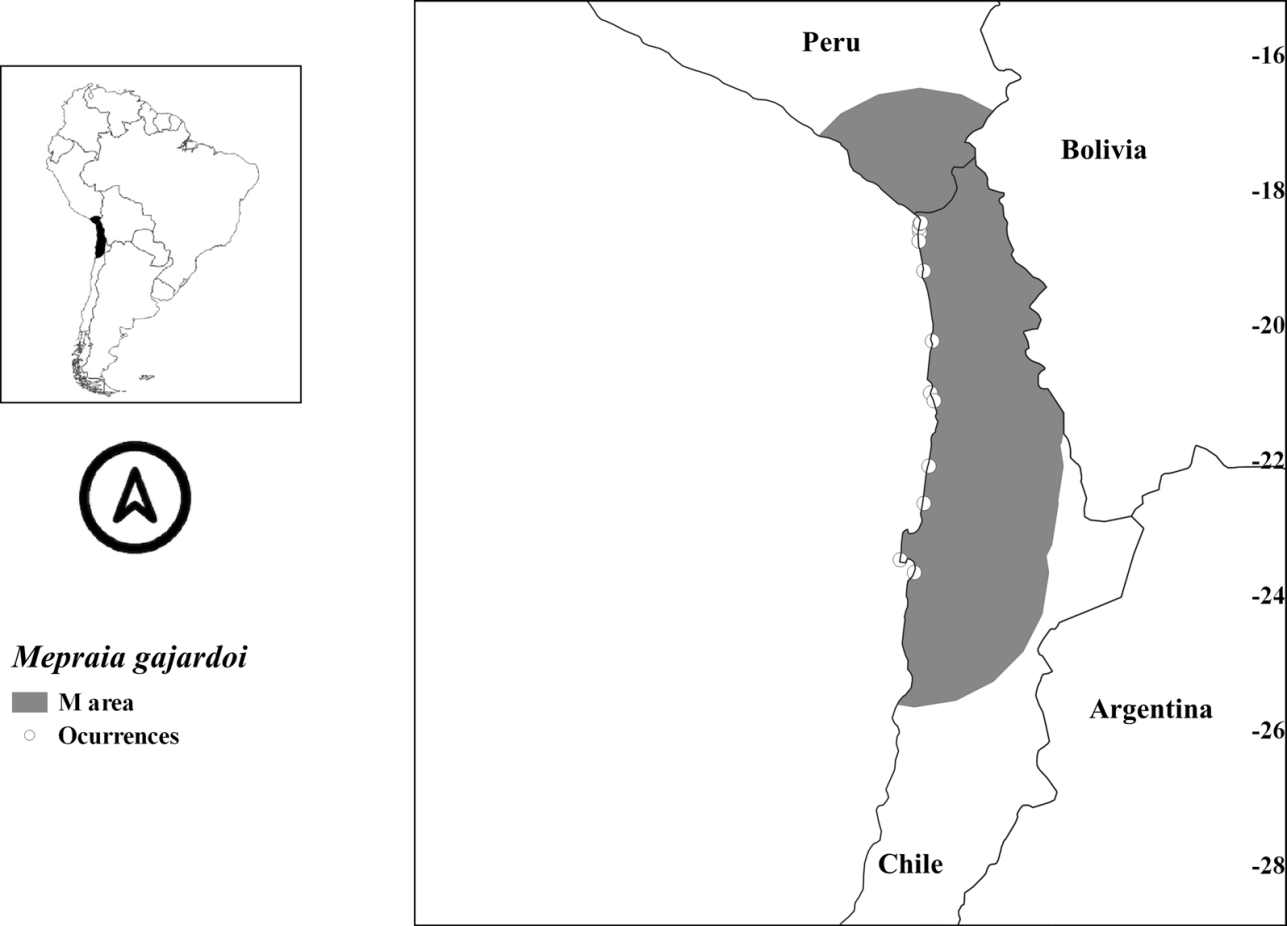
Accessible areas (M) and occurrences (open circles) used to model *Mepraia gajardoi*

Based on the hypothesis of accessible areas by dispersal over relevant time periods (M, hereafter) for both *Mepraia* species [15, 45], we set 2° (~222 km) buffer areas around each occurrence. The genus *Mepraia* is a dispersal restricted group, given that most developmental stages disperse by walking, unlike winged males that can also fly [4, 43, 46]; therefore, the accessible areas used were conservative compared to previous reports [e.g. 35, 37]. This buffer was constrained by the Andes and the Pacific Ocean, which constitute geomorphological features that *Mepraia* species would not naturally overcome [37]. These areas were used to extract the background data for modelling each species niche (Figs. 1, 2). We analysed the correlation among the selected variables within these calibration areas using 10,000 random points plus occurrences in R project v.3.4.1 (*Stats* and *corrplot* packages) [47].

The areas considered for projecting included the whole geographical area delimited by the administrative boundaries of Chile and Peru, using the same geomorphological criterion explained before [37]. For climate change projections, general circulation models (GCMs) were used, which resulted from the fifth phase of the Coupled Model Intercomparison Project CMIP5 [48], part of the Fifth Assessment Report (AR5) of the Intergovernmental Panel on Climate Change (IPCC) [49]. Each GCM exhibits different climate scenarios or representative concentration pathways (RCPs), which represent different ways of greenhouse gas concentrations resulting in radiative forcing, depending on human activity mitigation [50]. Scenarios are: (i) RCP 2.6, considered an optimistic scenario for maximum mitigation; (ii) RCP 4.5 and (iii) RCP 6.0, considered stabilization scenarios, in which there is increased radiative forcing but it stabilizes; and (iv) RCP 8.5, the most pessimistic scenario, in which higher levels of radiative forcing are recorded and the increase in temperature does not stop [50]. For future scenarios, we used five GCMs with the climatic projections for 2070: CCSM4 (CC, hereafter), GISS-E2-R (GS, hereafter), HadGEM-ES (HE, hereafter), IPSL-CM5A-LR (IP, hereafter) and MIROC-ESM (MR, hereafter). Each of the selected GCM has the four RCPs available [51]. These RCPs have 19 bioclimatic variables in raster format. The RCPs were obtained from climate model simulations planned as part of the World Climate Research Programme’s Fifth Coupled Model Intercomparison Project (CMIP5) [48, 50].

### Species distribution modelling

SDMs were constructed using Maxent v.3.3.3k [52, 53]. This software predicts species distribution, correlating sets of environmental predictors across a user-defined landscape that is divided into grid cells, with species occurrences [54]. Maxent is a machine-learning method that assesses the probability of a species distribution, by estimating a probability of distribution function of maximum entropy [53]. The method uses randomly selected pseudo-absences within an *a priori*-defined background area [55]. Maxent generally performs better than other software commonly used for SDMs; it has been widely used [53, 55, 56], and its ability to make predictions is supported when occurrence records are scarce [57, 58], as in this case. Maxent’s average performance was among the first algorithms tested in the Classic BAM (Biotic Abiotic Mobility) scenario and also for species with narrow niche breadth, but its results were variable depending on the measuring test [59]. No algorithm will be optimal under all circumstances because there is not a single best approach [59].

To run Maxent we used 10,000 background points, added samples to background, set auto features, and the regularization multiplier was set to 1. We allowed the creation of response curves, ran jackknife to measure variable importance (i.e. percent contribution and permutation importance [60]), activated Logscale raw/cumulative pictures, background predictions and verbose. Taking into account the number of occurrences of both species, cross-validation was replicated 15 times for *M. spinolai* and only three times for *M. gajardoi*; these numbers of replicates (k) corresponded to the number of independent subsets, used for training (k minus 1 subset), and evaluation of the model (using the remaining subset), with a maximum of 500 iterations. We used the average distribution map as output with logistic format for data values. Assuming niche conservation, the projections were performed using strict model transference between calibrated models and their current and projected future climate conditions. To this end, we deactivated clamping and extrapolation in Maxent, as recommended [61]. The performance of the models was evaluated using the area under the receiver operating characteristic curve (AUC) [53]. The AUC ranges from 0.5 for a model that performs no better than chance to 1.0 if the model fits perfectly. The AUC quantifies the degree to which the model identifies presences more accurately than random [53]. The constructed model was projected to current and future conditions.

We performed multivariate environmental similarity surface (MESS), which represents how similar a point is to a reference set of points with respect to a set of predictor variables. MESS provides an index of environmental similarity between each pixel and the median of the most dissimilar variable in M [62, 63]. We also performed mobility-oriented parity (MOP) analysis, which identifies areas of strict extrapolation and calculates environmental similarity between the calibration (M) and projection regions. MOP calculates multivariate distances from environmental variables associated with points across the projection region to a user-specified proportion of the environmental variables associated with points in the calibration region [63]; in our case, we used 50%. Both MESS and MOP analyses were performed in NicheToolBox [64].

For binary prediction of suitability (presence/absence) we used the minimum training presence threshold [65]; that is, the lowest predicted suitability value corresponding to an occurrence of each species in their respective M [66]. Once the binary predictions were obtained in QGIS Desktop v.2.18.19, we used two different approaches to calculate the suitability for each RCP. The first approach used the Boolean logic operator “OR”, allowing that if at least one of the GCM results for the same RCP resulted in a suitable cell, it was considered suitable for that RCP; this was the higher public health risk situation. The second approach involved the Boolean logic operator “AND”, forcing suitability in all five GCM results for the same RCP to retain the cell as suitable, conforming the lower public health risk situation. After this, we compared the resulting raster of the RCPs from each approach with the current potential distribution, obtaining two maps per RCP showing stable areas (present in the current potential distribution and in the resulting future climatic model), retraction areas (present in the current potential distribution but absent in the future projection) and expansion areas (absent in the current distribution but present in the modelled future scenario). The areas corresponding to each category were calculated using r.report tool in QGIS Desktop v.2.18.19. Maps were elaborated using publicly available shapefiles [67], our compiled occurrences, and the processed outputs of Maxent and NicheToolBox, in QGIS Desktop v.2.18.19. A complete summary of all used methods is depicted in a flow diagram included as Additional file 4: Figure S2.

## Results

### Variables in the models

The final model for *M. spinolai* included the five environmental variables mentioned above. The correlation coefficients are shown in Additional file 5: Table S2. Bio12 had a percent contribution of 60.6 and a permutation importance of 41.7, followed by Bio10 (30.8; 39.7), Bio7 (6.0; 9.9), Bio11 (4.0; 7.6) and Bio2 (1.2; 1.2). The final model for *M. gajardoi* included all the above variables except for Bio10, since this variable showed 0% of contribution in the first model (i.e. first run including the same variables used for *M. spinolai*), so it was not included in the following analyses. Bio2 had a percent contribution of 94.4 and a permutation importance of 37.6, followed by Bio11 (3.0; 21.3), Bio12 (2.2; 40.7) and Bio7 (0.4; 0.4). The correlation coefficients are shown in Additional file 5: Table S3 and the response curves’ figures for these variables are available in the repository.

### Validation of SDMs

The average AUC value for the model of *M. spinolai* was 0.878 with standard deviation 0.055. In the case of *M. gajardoi*, the average value of AUC was 0.984 and standard deviation 0.015.

### Extrapolation risk assessment

The resulting figures of MESS and MOP analyses are available in the repository, which show that there are no areas with strict extrapolation (i.e. with climate values outside the range of those in the calibration region). Both MESS and MOP for *M. gajardoi* showed a thin coastal band in the projection areas of Peru similar to its calibration area. In Chile, the similarity of the projection is concentrated in the north-central part of the country. For *M. spinolai*, both analyses showed more extended similar areas encompassing the Andes not including the Peruvian Amazonia. In Chile, the similarity areas showed a restriction in the northern part related to the desert, extending its distribution further south.

### Current and future SDMs for *M. spinolai* and *M. gajardoi*

The minimum suitability value of *M. spinolai* occurrences used as threshold for its binary prediction was 0.0126. All probability values below that number were considered non-suitable areas. The suitability area predicted under current climatic conditions for *M. spinolai* (317,580 km^2^) showed that the potential areas have similarities to its known distribution, including the inland valleys and, to a lesser extent, coastal areas and the Andes [4]. However, the current potential distribution extended to the north. This species showed an increased suitability inland in the Atacama Region but a reduction of suitability in the Atacama Desert, with some potential presence north in Peru (Fig. 1). It slightly extended south, encompassing approximately from 7° to 35°S.

The threshold value for binary prediction of *M. gajardoi* was 0.0407. All probability values above or equal to that number were considered suitable areas. The current potential distribution projected for *M. gajardoi* (42,727 km^2^) included its known distribution but extended through the coast of Peru. Figure 2 shows that it is a coastal species, with some inland suitable areas, but always near the coast, ranging from 8° to 27°S.

**Fig. 3:**
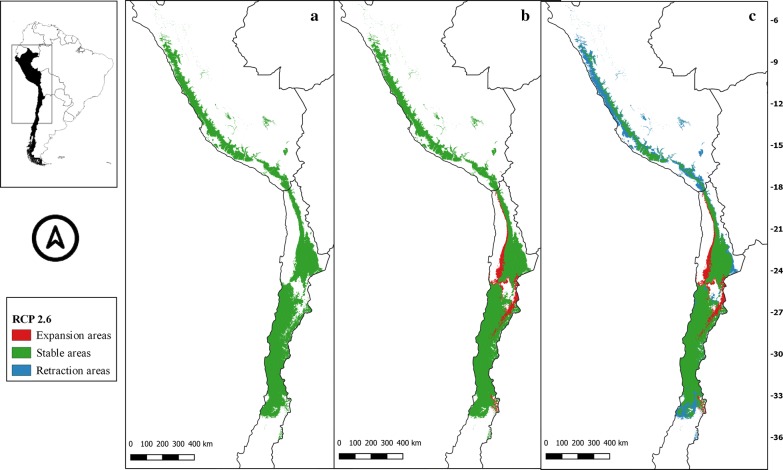
** a** Current potential distribution for *Mepraia spinolai*. Species model distribution projected as geographical distribution under future climate condition RCP 2.6 for 2070 in: a higher public health risk situation (**b**) and a lower public health risk situation (**c**). Stable (green), retraction (blue) and expansion (red) areas are shown for each public health risk situation. On the left, reference map of South America showing the projection areas. On the right, latitude is shown

**Fig. 4:**
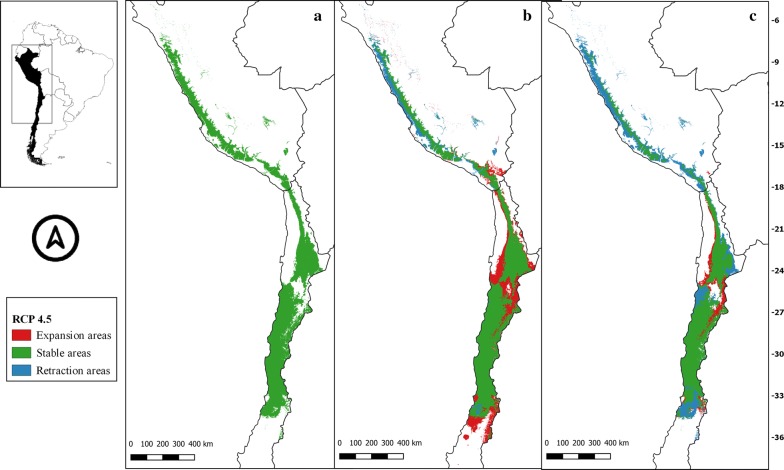
** a** Current potential distribution for *Mepraia spinolai*. Species model distribution projected as geographical distribution under future climate condition RCP 4.5 for 2070 in: a higher public health risk situation (**b**) and a lower public health risk situation (**c**). Stable (green), retraction (blue) and expansion (red) areas are shown for each public health risk situation. On the left, reference map of South America showing the projection areas. On the right, latitude is shown

**Fig. 5:**
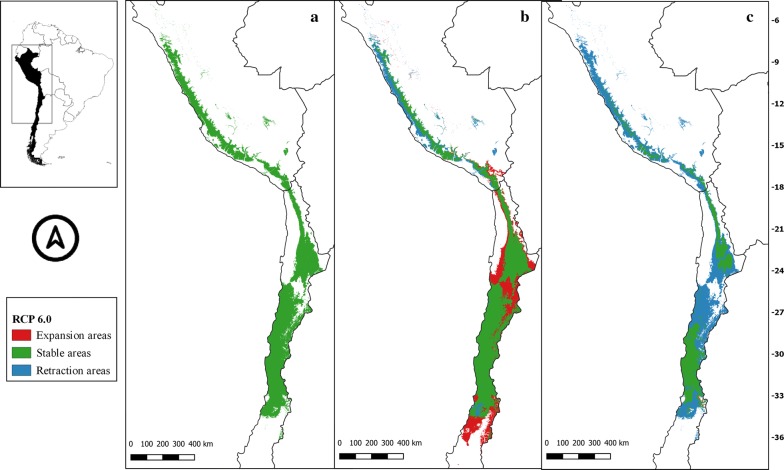
** a** Current potential distribution for *Mepraia spinolai*. Species model distribution projected as geographical distribution under future climate condition RCP 6.0 for 2070 in: a higher public health risk situation (**b**) and a lower public health risk situation (**c**). Stable (green), retraction (blue) and expansion (red) areas are shown for each public health risk situation. On the left, reference map of South America showing the projection areas. On the right, latitude is shown

**Fig. 6:**
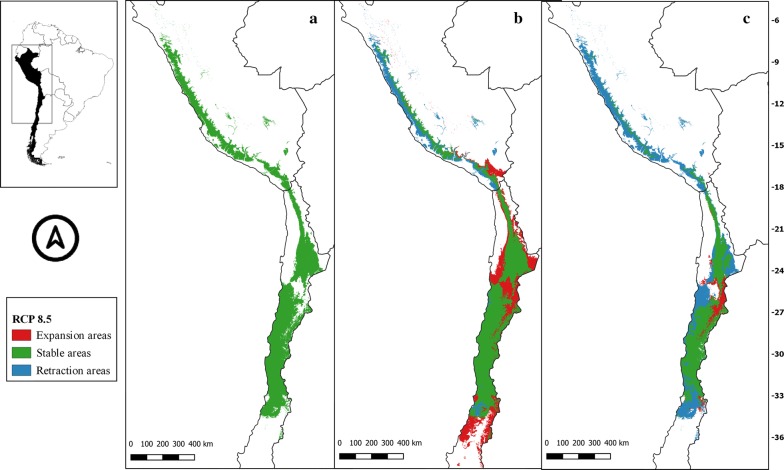
** a** Current potential distribution for *Mepraia spinolai*. Species model distribution projected as geographical distribution under future climate condition RCP 8.5 for 2070 in: a higher public health risk situation (**b**) and a lower public health risk situation (**c**). Stable (green), retraction (blue) and expansion (red) areas are shown for each public health risk situation. On the left, reference map of South America showing the projection areas. On the right, latitude is shown

**Fig. 7:**
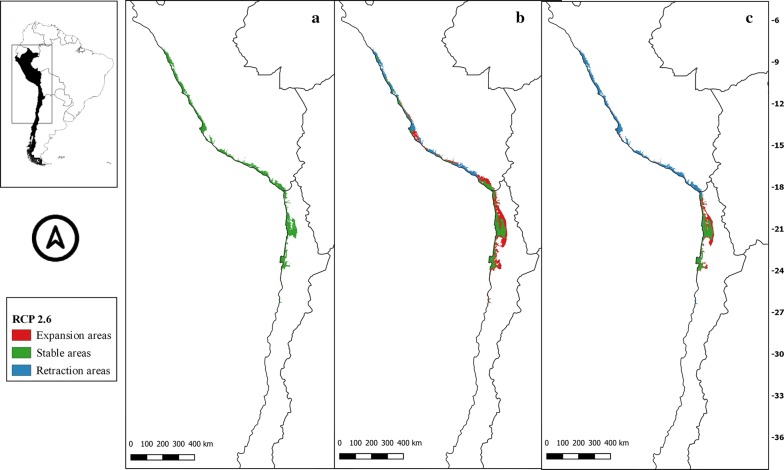
** a** Current potential distribution for *Mepraia gajardoi*. Species model distribution projected as geographical distribution under future climate condition RCP 2.6 for 2070 in: a higher public health risk situation (**b**) and a lower public health risk situation (**c**). Stable (green), retraction (blue) and expansion (red) areas are shown for each public health risk situation. On the left, reference map of South America showing the projection areas. On the right, latitude is shown

**Fig. 8:**
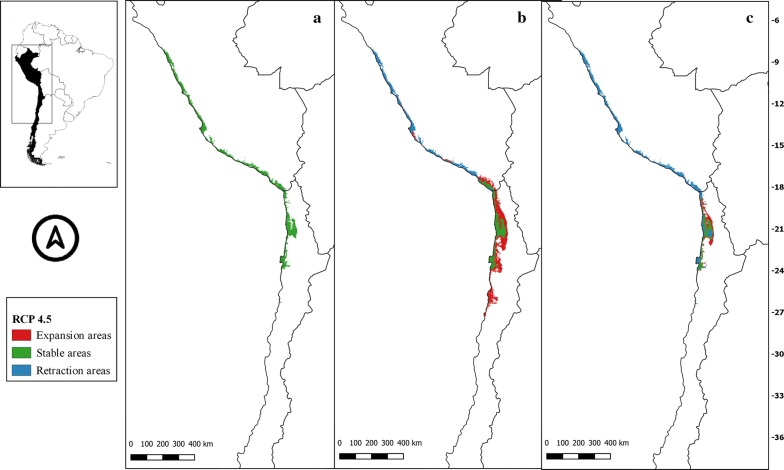
** a** Current potential distribution for *Mepraia gajardoi*. Species model distribution projected as geographical distribution under future climate condition RCP 4.5 for 2070 in: a higher public health risk situation (**b**) and a lower public health risk situation (**c**). Stable (green), retraction (blue) and expansion (red) areas are shown for each public health risk situation. On the left, reference map of South America showing the projection areas. On the right, latitude is shown

**Fig. 9:**
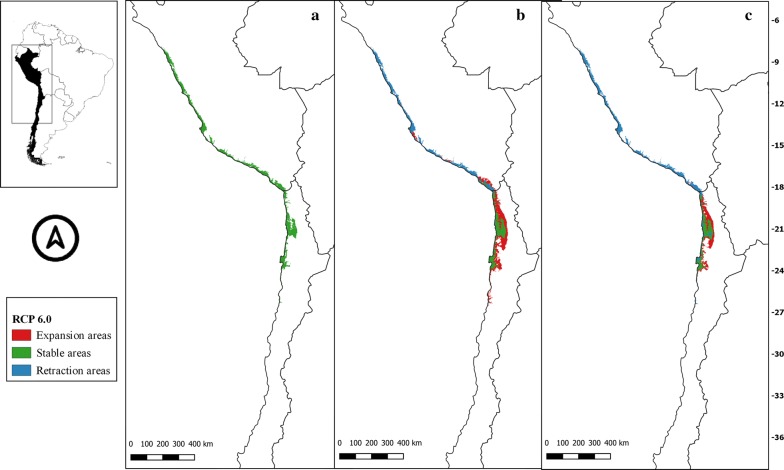
** a** Current potential distribution for *Mepraia gajardoi*. Species model distribution projected as geographical distribution under future climate condition RCP 6.0 for 2070 in: a higher public health risk situation (**b**) and a lower public health risk situation (**c**). Stable (green), retraction (blue) and expansion (red) areas are shown for each public health risk situation. On the left, reference map of South America showing the projection areas. On the right, latitude is shown

**Fig. 10:**
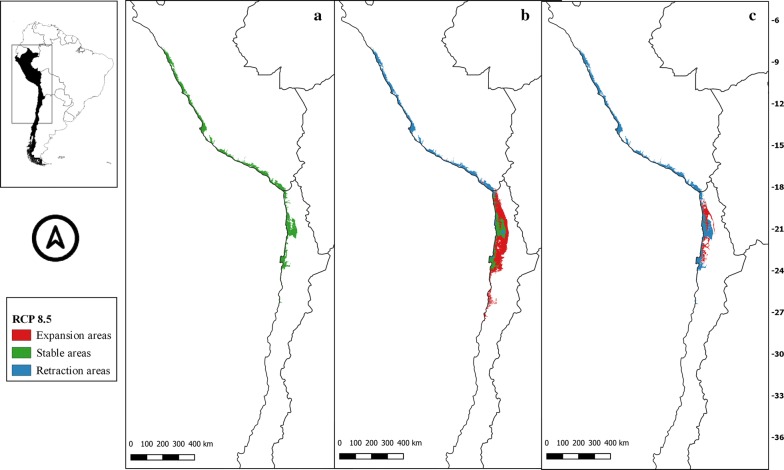
** a** Current potential distribution for *Mepraia gajardoi*. Species model distribution projected as geographical distribution under future climate condition RCP 8.5 for 2070 in: a higher public health risk situation (**b**) and a lower public health risk situation (**c**). Stable (green), retraction (blue) and expansion (red) areas are shown for each public health risk situation. On the left, reference map of South America showing the projection areas. On the right, latitude is shown

The suitability areas in all future climate scenarios for *M. spinolai* and *M. gajardoi* are shown in Figs. 3, 4, 5, 6, 7, 8, 9 and 10, respectively. Tables 1 and 2 show in detail the stable, retraction and expansion areas in future scenarios, compared to the current potential distribution of *M. spinolai* and *M. gajardoi*, respectively.

**Table 1:** Potential suitability area (km^2^) of *Mepraia spinolai* for each climate scenario

Public health situation	Scenario	Stable area	Retraction area	Expansion area	Total potential suitable area
Higher risk	2.6	287,092	30,488	102,008	389,100
	4.5	275,111	42,468	107,339	382,450
	6.0	268,577	49,003	109,677	378,254
	8.5	254,585	62,995	134,430	389,015
Lower risk	2.6	231,673	85,906	29,034	260,707
	4.5	208,307	109,273	28,889	237,196
	6.0	104,814	212,766	647	105,461
	8.5	161,338	156,242	24,694	186,032

**Table 2:** Potential suitability area (km^2^) of *Mepraia gajardoi* for each climate scenario

Public health situation	Scenario	Stable area	Retraction area	Expansion area	Total potential suitable area
Higher risk	2.6	24,671	18,056	23,911	48,582
	4.5	19,345	23,382	35,104	54,449
	6.0	16,889	25,838	27,274	44,163
	8.5	13,121	29,606	35,959	49,080
Lower risk	2.6	14,951	27,777	8337	23,288
	4.5	9388	33,339	7888	17,276
	6.0	12,979	29,749	14,784	27,763
	8.5	369	42,359	9812	10,181

For *M. spinolai* in the future higher risk public health situation, the different RCPs showed similar potential distributions, maintaining most of its known distribution, but with new areas expanding this species range to the north to the arid diagonal of the Atacama Desert (between 24° and 27°S) and to the south (*c.*36°S), with some retraction in the coast of Peru. On the other hand, the lower risk situation showed a retraction of potential suitable areas, with higher losses in the 6.0 scenario (Table 1). Retraction occurred to the south in all scenarios modelled, losing more areas while increasing the scenarios’ severity. The arid diagonal area appears to be a barrier to dispersion, showing retraction areas in all scenarios except for RCP 2.6. The current potential distribution in Peru was reduced in all scenarios but in different magnitudes (Figs. 3, 4, 5, 6).

The potential higher risk situation in the distribution of *M. gajardoi* in the future showed some new suitable inland areas near the coast that seem to be consistent among RCP, and other potential future distribution areas further south of its known distribution by the coast. In general, the potential future distributions in the four scenarios were similar to the current potential suitable area but showed retraction in the potential areas by the coast of Peru, especially in the most pessimistic scenario (RCP 8.5). In the lower risk situation, this retraction tendency in Peru was evident, even in the most optimistic scenario (RCP 2.6). An overall reduction trend of potential suitable Chilean areas was detected in all scenarios in the lower risk situation (with different magnitudes regarding RCP), showing a substantial loss of suitable areas in both countries in the most pessimistic scenario (Figs. 7, 8, 9, 10).

## Discussion

In this study, we collected data from different sources on the occurrence of *M. spinolai* and *M. gajardoi*, endemic triatomine species from Chile, to assess their current climate suitability and predict their potential distribution under future climate scenarios. Both models performed significantly different than chance.

The SDM built for *M. spinolai* was mainly associated with annual precipitation (Bio12) and mean temperature of the warmest quarter (Bio10), both contributing with more than 90% in the model. Precipitation above 400 mm decreased its occurrence probability, with the maximum suitability values about 100 mm. High rainfall might be creating an excessively humid microenvironment promoting entomopathogenic fungus growth [68]; therefore, this would probably decrease vector fitness. On the other hand, moderate precipitation may be indirectly related to the presence of *M. spinolai*, because rainfall promotes primary productivity (i.e. plant communities) and this, in turn, positively affects the abundance of small rodent species such as *Octodon degus* and *Phyllotis darwini*, hosts of *M. spinolai* [69, 70]. Then, the increase in host populations would have a positive effect on vector population demographic parameters.

Mean temperature of the warmest quarter is probably relevant because *M. spinolai* inhabits areas with marked seasonality that affects this ectothermic species, forcing reproduction during the warm season [7, 71]. Temperature is related to triatomine dispersal to locate hosts and mates, and diapause induction [44, 47]. The maximum suitability for this variable was at 24 °C; however, under 10 °C and above 24 °C, suitability drops abruptly. The suitability maps for *T. infestans* in Chile and Peru [34] are quite similar to ours for *M. spinolai*. Temperature annual range, mean diurnal range and mean temperature of the coldest quarter also contributed to *M. spinolai* model, showing similarities to the models reported for several triatomine species, including *T. infestans* [34].

Regarding the suitability areas shown in the model, the current potential distribution of *M. spinolai* showed two main areas: one limited to semiarid areas from 27° to 34°S, and another from 8° to 27°S including the Atacama Desert and some areas of Peru. The first area includes most of the known distribution of this species [72]. In the second main area, no *M. spinolai* occurrences have been notified, but it is a suitable climate zone to invade according to our model. The arid diagonal of the Atacama Desert has probably prevented its colonization. Given that *T. infestans* colonized houses in this northern area [27] and considering that these two species have been detected coexisting under the same sylvatic environmental conditions [5], it is plausible that *M. spinolai* could be currently present but undetected or could eventually invade this area [73, 74]. In fact, our current potential distribution model for *M. spinolai* in Chile is very similar to that recently modelled for *T. infestans* in this country [27].

The model of *M. gajardoi* was mainly related to mean diurnal range (Bio2), which is the mean of the monthly minimum temperature subtracted from the maximum temperature, showing a decrease in the suitability under wide thermal oscillation. Then, the model is explained mainly by temperature. The current potential distribution of *M. gajardoi* included mainly coastal areas from 8° to 27°S, characterized by the Pacific anticyclone effect that prevents most rainfall and high temperature differences [75]. Therefore, this vector species would be adapted to areas with scarce thermal and humidity fluctuations, preventing its spread to the Atacama Desert, characterized by wide daily thermal variations and excessive dryness.

When analysing the overlap between the current potential distribution from both species, we detected an overlap area encompassing around 24°40′ to 26°35′S by the coast, an area presenting another *Mepraia* species, *M. parapatrica*. In this zone, it has been reported that climatic and ecologic characteristics are intermediate from those where *M. spinolai* and *M. gajardoi* inhabit [45]. In our study, we confirm that the climate of this area would be suitable for both *M. gajardoi* and *M. spinolai*, without discarding that hybridization could be occurring [76]. There is another area where both species’ current potential distributions overlap, mainly near the coast of Peru, but neither of these species have been reported. We suggest that this area should be sampled, to assess if any member of the *Mepraia* genus are present, especially considering that this area is currently colonized by triatomine species from the *Triatoma* and *Panstrongylus* genus [77]. Therefore, the ecological conditions, including biotic and abiotic factors, might be conducive for triatomine colonization.

Regarding RCPs 2.6, 4.5, 6.0 and 8.5, it is predicted that the temperature will increase an average of 1.0, 1.8, 2.2 and 3.7 °C, respectively [49]. For Chile, the increase in temperature under a severe scenario will be modest in the coast (0.5 to 1.0 °C) but will increase towards the Andes, where it may rise up to 5 °C in north and central Chile [78]. It is expected that these arid and semi-arid zones (that encompass most of the potential distribution areas of these species) will show modest precipitation changes, but the south will present a significant reduction in rainfall and an increase in temperature [78]. These climate modifications would promote the future expansion of the potential distribution of *M. spinolai*. For the coastal areas used by *M. gajardoi*, low thermal oscillation and scarce precipitation change are expected in the future [78]. A previous study has shown that insect species living at higher latitudes, as our study subjects, have broader thermal tolerance and live in climate cooler than their physiological optimal; therefore, climate warming may enhance their fitness performance [79]. Our results for both *M. spinolai* and *M. gajardoi* show an increase in all future scenarios when modelling the higher public health risk situation, and a reduction in the suitable areas when modelling the lower risk situation, so there is consistency in the approaches utilized. Transmission risk models are useful and predictive if they can anticipate the effects of climate changes on spatial risk patterns, so this detected uncertainty must be taken into account in future predictions [14]. In our study, we considered health risk using vector distribution modelling according to climate. To be more informative for public health risk assessments, future studies should include information on vector competence and/or infection prevalence, in case these parameters are affected by climate.

In this study, we used interpolated climate data. These data were obtained from available climate stations; however, the density of these is relatively low in the study area and therefore these interpolated data may not necessarily reflect its exact climate, particularly in mountainous areas [41]. Remotely sensed data may be more informative than ground-based data sets [14]; in our case, in spite of its limitations, we used the Worldclim dataset because it includes future projections needed for future modelling, but for new studies of the potential current distribution of these vectors, data from remote sensors may be preferable. Our projections consider climate at a regional scale, but it is well known that microrefugia are present (i.e. sites that support locally favourable climate within unfavourable regional climate), which may allow populations to persist outside their main distributions [80]. Then, it is possible our modelled distributions could be constrained and, therefore, underestimated.

A limitation of our models relates to their validation. To construct the SDMs, we used all the occurrences reported in the literature and all those provided by colleagues and the Ministry of Health; therefore, we were unable to validate them with additional independent occurrences (locations not used for modelling). Instead, we relied on the species identification accuracy, ensuring no false positive records were included as occurrences. Even though the performance of our models was evaluated by the AUC, a controversial metric for climatic niche modelling, we consider our results are relevant in terms of current and future public health risk. In addition, MESS and MOP analyses were used to minimize extrapolation errors to assess predictions [63]. In our study, the suitability areas in the models were mostly restricted to environmental zones similar to the calibration areas of each species; therefore, supporting our models.

The areas indicated as suitable in the current potential distribution could be inspected to evaluate if these species are currently present. However, the lack of species presences could be the result of constraints in their connectivity to present or past populations, parameter directly dependent on dispersal [37]. Both *Mepraia* species present adult alary polymorphism [4], so their ability to surpass unsuitable areas to reach further suitable ones would be impaired, compared to other winged triatomine species. This would prevent colonization when suitable patches are not contiguous unless they are passively transported, as occurred with *T. infestans* [81]. An additional constraint to occupancy is microhabitat quality. These sylvatic species require rocky outcrops, bromeliads or rock crevices to establish, with the appropriate vertebrate feeding sources to maintain triatomine colonies [4, 8, 10, 13, 43, 70, 82, 83]. One last consideration when sampling to detect vectors in the field is the sensitivity of the used methods. Assuming detection is imperfect, areas could be determined as negative when in fact they may contain triatomines, so this should also be considered for model validation [73]. This was not an issue in the construction of our models, because Maxent only requires presences as input. However, occurrences’ sampling bias may have partially influenced our results, as we cannot assure that the whole accessible areas were sampled to obtain the input occurrences for this study, and we did not explicitly model the probability of sampling a location in the calibration areas [54]. Future studies should consider all these biases when projecting species distributions.

We assume that the construction of our models considered all the relevant climatic variables for *Mepraia* species; however, at this point we cannot discard that relevant variables for these SDMs were overlooked. Future studies on these vector species should also consider physiological data to predict species ranges. In this study, we used two approaches to assess potential suitability areas in the future. The lower risk situation is a conservative approach, because it would not overestimate but could underestimate the future potential distribution of both species. Conversely, the higher risk situation could show the opposite limitation. Furthermore, current weather observations (of 30 years as minimum) have not been compared with the baseline of the GCMs, at least for Chile, to assess which model fits better for this region. To account for this caveat, we included the results of five different GCMs for each RCP. Currently, species of the genus *Mepraia* seem to be constrained by the Pacific Ocean to the west and the Andes mountain range to the east. In these areas, several different climates are present in short stretches of land. For example, the area from 18° to 25°S is subdivided into three types of climates: coastal desert, interior desert and high desert [84], implying that climate change projections in face of this geographical complexity may present strong representation problems.

Finally, if triatomines conserve their climatic niche into the future, i.e. niche conservatism, species ought to migrate or become extinct when facing adverse conditions [22]. However, other alternatives must be considered, whether species can modify its climatic niche due to phenotypic plasticity or adaptive microevolution [85]. In our study that topic was not covered, but future studies should consider the fact that phenotypic plasticity can be an attenuating factor of climate change effects, and assist in short- and long-term adaptation [86, 87].

## Conclusions

The climate requirements of *M. spinolai* are mostly related to annual precipitation and mean temperature of the warmest quarter. Its potential distribution was projected to semi-arid and Mediterranean climates, fitting the known distribution of this species, located in inland valleys of arid and semiarid climate and, to a lesser extent, along the coast. The SDM of *M. gajardoi* was built mainly with mean temperature diurnal range. The potential distribution of *M. gajardoi* was limited to areas with coastal desert climate of Chile and Peru. Additional sampling and surveillance are required to corroborate if these vectors are inhabiting areas detected as currently suitable by our models but still have no reports of their presence. Under climate change scenarios, *M. spinolai* and *M. gajardoi* would mainly conserve their current distributions, and expand to zones with similar climates. However, the biotic features (e.g. host and refuge availability) in these areas could be restricting factors that will need to be considered when generating vector control programs. Prevention campaigns should be anticipated in areas where historically no wild triatomines have been reported, and control measures must be established and/or reinforced in areas where these triatomines are expected to persist.

## Supplementary information

**Additional file 1: Figure S1.** Ecoregions present in the study area, which includes Chile and Peru.

**Additional file 2: Dataset S1.** Complete dataset for *Mepraia spinolai* occurrences. **Dataset S2.** Complete dataset for *Mepraia gajardoi* occurrences.

**Additional file 3: Table S1.** List of articles used to obtain occurrence coordinates for *Mepraia spinolai* and *Mepraia gajardoi*.

**Additional file 4: Figure S2.** Flow diagram of the methods followed in this study.

**Additional file 5: Table S2.** Correlation matrix for bioclimatic variables used to model *Mepraia spinolai* potential distribution. **Table S3.** Correlation matrix for bioclimatic variables used to model *Meparaia gajardoi* potential distribution.

## Data Availability

Data supporting the conclusions of this article are included within the article and its additional files. The data generated and/or analysed during the present study are available in the Figshare repository, https://figshare.com/s/79896af2b019b2eac0a7.

## Abbreviations

RCP: representative concentration pathways
SDM: species distribution model
IPCC: Intergovernmental Panel on Climate Change
GCM: general circulation models
CMIP5: Fifth phase of the Coupled Model Intercomparison Project
AR5: Fifth Assessment Report
AUC: area under the curve
