# Supplementary figures and images for: Potential impact of climate change on the geographical distribution of two wild vectors of Chagas disease in Chile: Mepraia spinolai and Mepraia gajardoi
Source: Parasit Vectors. 2019 Oct 14;12:478. doi: 10.1186/s13071-019-3744-9 (PMC6792221; doi:10.1186/s13071-019-3744-9)

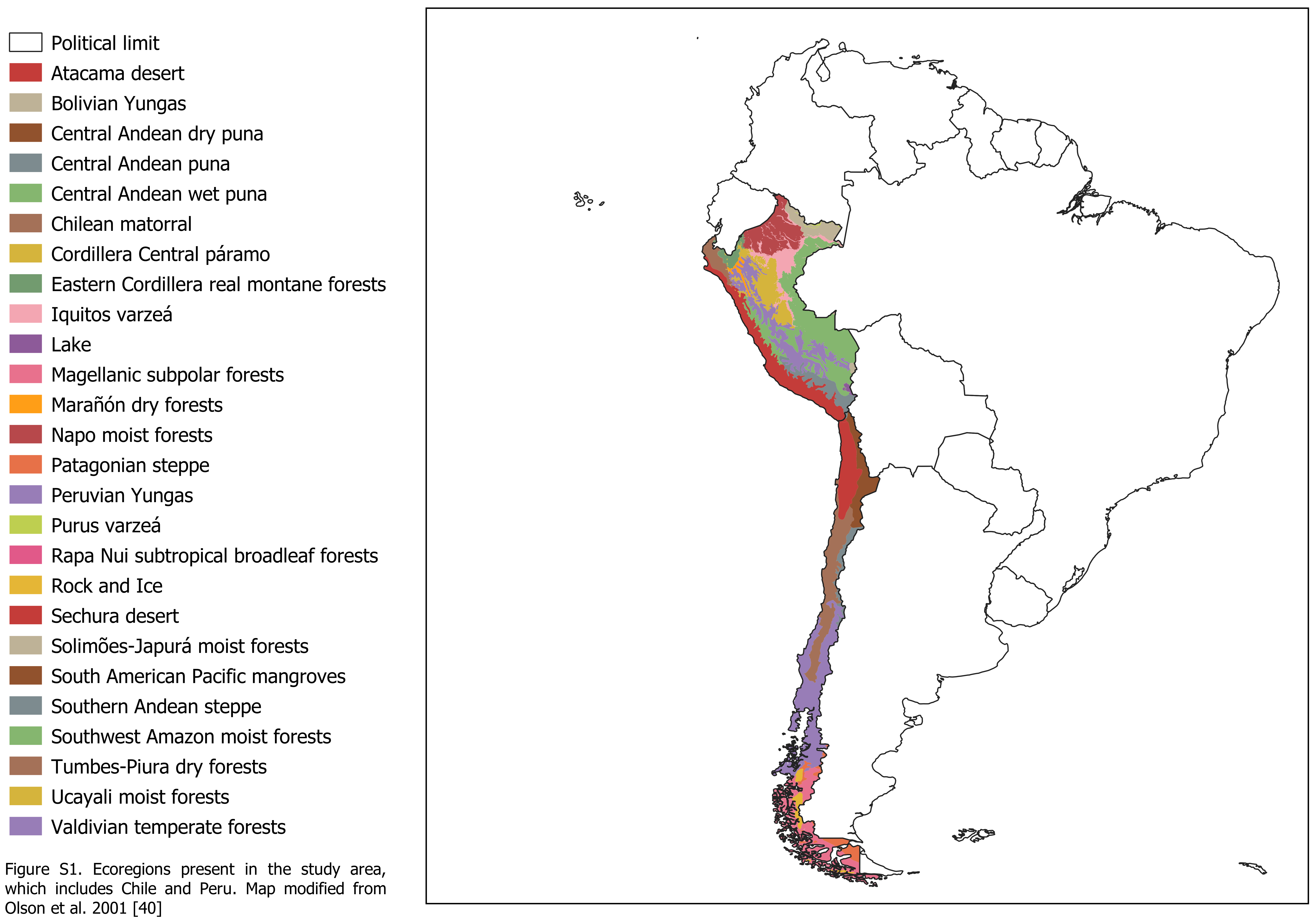

Supplement: Supplementary file 1 — Additional file 1: Figure S1. Ecoregions present in the study area, which includes Chile and Peru. [file 13071_2019_3744_MOESM1_ESM.tif]

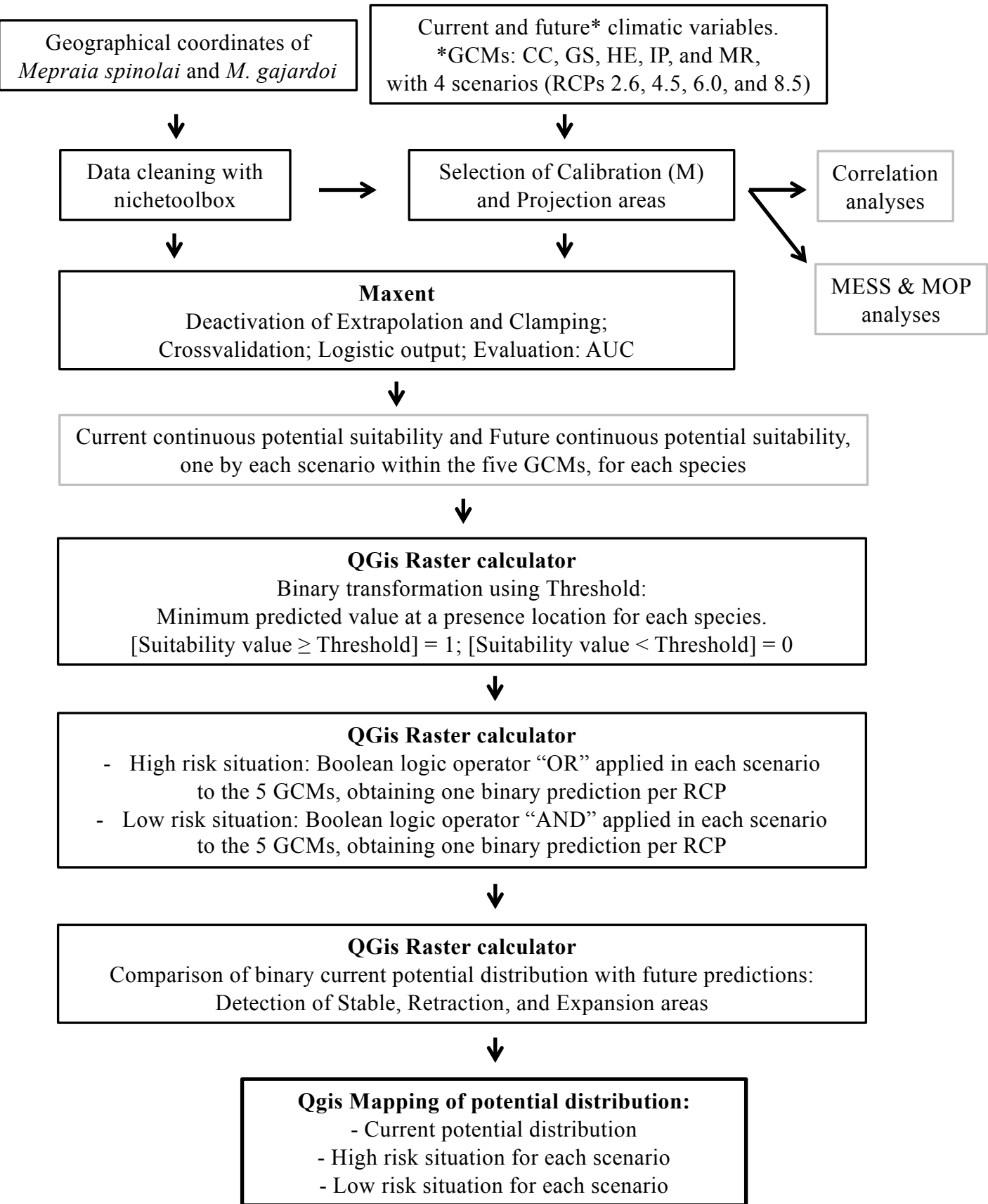

**Figure S4.** Flow diagram of the methods followed in this study.

Supplement: Supplementary file 4 — Additional file 4: Figure S2. Flow diagram of the methods followed in this study. [file 13071_2019_3744_MOESM4_ESM.pdf]
