# Supplementary material for: Potential impact of climate change on the geographical distribution of two wild vectors of Chagas disease in Chile: Mepraia spinolai and Mepraia gajardoi
Source: Parasit Vectors. 2019 Oct 14;12:478. doi: 10.1186/s13071-019-3744-9 (PMC6792221; doi:10.1186/s13071-019-3744-9)
Supplement: Supplementary file 3 — Additional file 3: Table S1. List of articles used to obtain occurrence coordinates for Mepraia spinolai and Mepraia gajardoi. [file 13071_2019_3744_MOESM3_ESM.docx]

**Additional file 3: Table S1.** List of articles used to obtain occurrence coordinates for *M. spinolai* and *M. gajardoi*.

| References |
| --- |
| Bacigalupo A, et al. Rev Med Chil. 2006;134:1230−6. |
| Bacigalupo A, et al. Mem Inst Oswaldo Cruz. 2010;105:633−41. |
| Botto-Mahan C et al. Acta Trop. 2002;82:377−80. |
| Botto-Mahan C, et al. Acta Trop. 2006;98:219−23. |
| Botto-Mahan C, et al. Acta Trop. 2008;105:166−9. |
| Calleros L, et al. Infect Genet Evol. 2010;10:221−8. |
| Campos R, et al. Infect Genet Evol. 2011;11:329−33. |
| Campos C, et al. Infect Genet Evol. 2013;19:280−6. |
| Canals M, et al. Mem Inst Oswaldo Cruz. 1999;94: 687-92. |
| Carvajal A, et al. Parasitol. Latinoam. 2007;62:118–21. |
| Cattan PE, et al. Mem Inst Oswaldo Cruz. 2002;97:285−7. |
| Egaña C, et al. Am J Trop Med Hyg. 2014;91:534-6 |
| Frías DA, et al. Rev Chil Hist Nat. 1998;71:177−88. |
| Frías-Lasserre D. Neotrop Entomol. 2010;39:572−83. |
| Moreno M, et al. Infect Genet Evol. 2005;6:228−34. |
| Sagua F, et al. Mem Inst Oswaldo Cruz. 2000;95:167−70. |
| Schaub GA, et al. Parasitenkunde 1984;70:3-9. |
| Schofield CJ, et al. Med Vet Entomol. 1998;12:30–8.  Toledo A, et al. Am J Trop Med Hyg. 2012;88:285−8.  Venegas J, et al. Parasitol. 1997;115:41−6. |
