# Supplementary material for: Potential impact of climate change on the geographical distribution of two wild vectors of Chagas disease in Chile: Mepraia spinolai and Mepraia gajardoi
Source: Parasit Vectors. 2019 Oct 14;12:478. doi: 10.1186/s13071-019-3744-9 (PMC6792221; doi:10.1186/s13071-019-3744-9)
Supplement: Supplementary file 5 — Additional file 5: Table S2. Correlation matrix for bioclimatic variables used to model Mepraia spinolai potential distribution. Table S3. Correlation matrix for bioclimatic variables used to model Meparaia gajardoi potential distribution. [file 13071_2019_3744_MOESM5_ESM.docx]

**Additional file 5: Table S2.** Correlation matrix for bioclimatic variables used to model *Mepraia spinolai* potential distribution. Correlation values and statistical significance are shown above and below the diagonal, respectively. Bio2: mean temperature diurnal range, Bio7: temperature annual range, Bio10: mean temperature of warmest quarter, Bio11: mean temperature of coldest quarter, Bio12: annual precipitation. ***: *P* < 0.001. n = 10150.

| Variables | Bio2 | Bio7 | Bio10 | Bio11 | Bio12 |
| --- | --- | --- | --- | --- | --- |
| Bio2 |  | 0.69 | -0.25 | -0.37 | 0.30 |
| Bio7 | *** |  | 0.13 | -0.21 | 0.81 |
| Bio10 | *** | *** |  | 0.93 | 0.18 |
| Bio11 | *** | *** | *** |  | -0.15 |
| Bio12 | *** | *** | *** | *** |  |

**Additional file 5: Table S3.** Correlation matrix for bioclimatic variables used to model *Meparaia gajardoi* potential distribution. Correlation values and statistical significance are shown above and below the diagonal, respectively. Bio2: mean temperature diurnal range, Bio7: temperature annual range, Bio10: mean temperature of warmest quarter, Bio11: mean temperature of coldest quarter, Bio12: annual precipitation. ***: *P* < 0.001. n = 10013.

| Variables | Bio2 | Bio7 | Bio10 | Bio11 | Bio12 |
| --- | --- | --- | --- | --- | --- |
| Bio2 |  | 0.94 | -0.85 | -0.84 | 0.53 |
| Bio7 | *** |  | -0.82 | -0.87 | 0.45 |
| Bio10 | *** | *** |  | 0.98 | -0.76 |
| Bio11 | *** | *** | *** |  | -0.68 |
| Bio12 | *** | *** | *** | *** |  |
